# A Huge Hemangioma of the Urinary Bladder: A Case Report and Literature Review

**DOI:** 10.7759/cureus.52852

**Published:** 2024-01-24

**Authors:** Muhannad Wael, Wael Abuarafeh, Mohammad N Ghneimat, Murad Al Hammouri, Mutaz W Abuarafeh, Ahmad M Nabali

**Affiliations:** 1 Urology, Saint Joseph Hospital, Jerusalem, PSE; 2 Faculty of Medicine, Al-Quds University, Jerusalem, PSE; 3 Internal Medicine, Saint Joseph Hospital, Jerusalem, PSE

**Keywords:** hemangioma, bladder cancer, partial cystectomy, urinary bladder, cavernous hemangioma

## Abstract

Cavernous hemangioma of the bladder is a benign and very rare vascular tumor. It can be isolated or part of a syndrome. Neither clinical symptoms nor imaging modalities lead to a definitive diagnosis as there are no specific findings on imaging studies or specific symptoms. Painless gross hematuria is the most common chief complaint and presentation and should never be underestimated.

Here, we report a case of a large hemangioma of the urinary bladder in a young man who presented with recurrent recent episodes of painless gross hematuria and, surprisingly, with a previous episode of painless hematuria 15 years ago, which was treated successfully with partial cystectomy. We discuss the clinical features, evaluation, diagnosis, biopsy, management, the challenges encountered in treatment, and assert the value of tissue diagnosis and follow-up pattern and period. Due to the rarity of the tumor and lack of trials and evidence-based guidelines for management, treatment modalities vary and the risk for recurrence is questionable and not known.

## Introduction

Cavernous hemangioma of the bladder (CHB) is a benign vascular tumor. Hemangioma of the urinary bladder is very rare, accounting for only 0.6% of all bladder tumors [1]. There have been fewer than 100 reported cases of histologically proven hemangioma of the urinary bladder [2]. The diagnosis can be difficult as imaging features are non-specific and the differential diagnosis is broad [3].

There are no specific clinical features or symptoms except for gross painless hematuria. Moreover, imaging findings are non-specific unless describing either a solid lesion or wall thickening which is mostly hypervascularized and calcified. Cautious and precise biopsy and immunohistochemistry tests are needed for an accurate diagnosis.

Because of its rarity, the diagnosis and treatment of hemangioma of the urinary bladder are tricky. Diagnosis of bladder hemangioma is challenging due to the vague and non-specific clinical symptoms and findings on imaging investigations. As cystoscopy alone is not enough for accurate diagnosis, tissue biopsy is needed for a definite diagnosis, which should be taken from the vascularized area of the mass. Careful histopathology and immunohistochemistry are required to establish the correct diagnosis [4].

The predominant clinical symptom of urinary bladder hemangioma is the painless recurrence of isolated gross macroscopic hematuria with or without irritative urinary symptoms and abdominal pain [5]. Gross hematuria should be evaluated by ultrasonography as a non-invasive first-line method followed by computed tomography (CT) and magnetic resonance imaging (MRI) for precise details of tumor location and extension.

## Case presentation

A healthy 21-year-old male known to have a huge urinary bladder hemangioma was referred to our clinic for further evaluation and management with consideration of partial cystectomy. The patient presented with recent recurrent episodes of painless gross hematuria and complained of frequent urination, a sensation of incomplete voiding, and occasional lower abdominal discomfort for one year. The patient provided a history of a single episode of painless gross hematuria at the age of six years which resolved spontaneously and never recurred. He was a smoker for four years, with no history of exposure to any agents considered to be risk factors for bladder cancer.

The physical examination did not show any palpable mass or enlarged lymph nodes. Hematological and biochemical parameters were within the normal range. This patient’s renal function test (blood urea nitrogen and creatinine) was within normal ranges at presentation.

An initial ultrasound showed a 6 × 4 cm mass lesion arising from the anterior wall of the urinary bladder. A cystoscopy done at another hospital revealed a large solid mass, bluish in color, measuring about 7 cm, mainly in the anterior wall and extending to the dome of the bladder. A punch biopsy was obtained, and the histopathological examination revealed a cavernous hemangioma.

**Figure 1:**
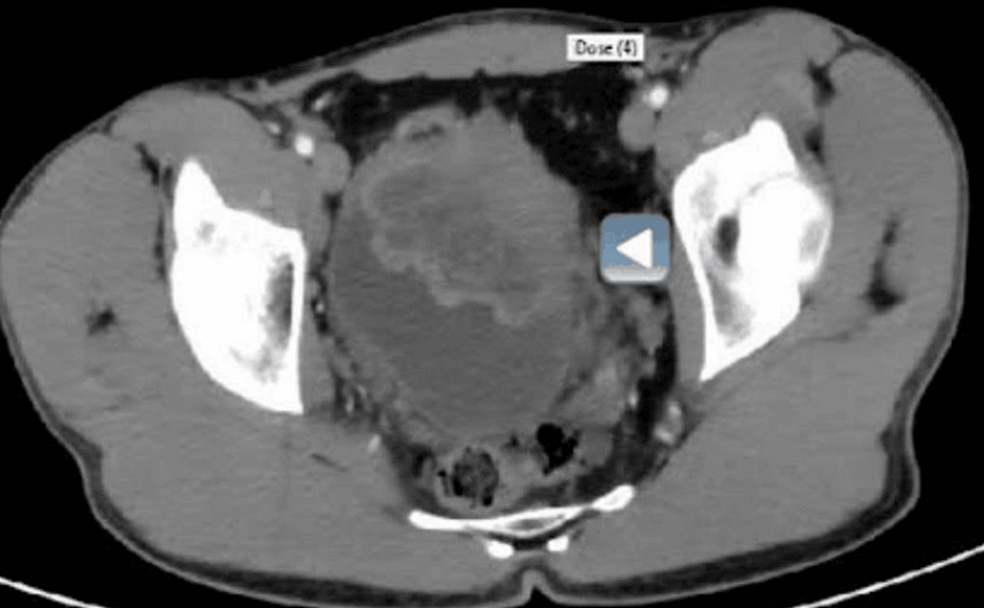
Enhanced CT scan of the abdomen and pelvis showing a large anterior urinary bladder mass lesion with luminal filling of the anterior compartment of the urinary bladder and mural involving the anterior wall extending beyond the urinary bladder, measuring about 7.5 × 6 × 6 cm in the mediolateral, anteroposterior, and craniocaudal dimensions, respectively.

In our hospital, as shown in Figure 1, a CT scan of the abdomen and pelvis showed a large anterior urinary bladder mass lesion with luminal filling of the anterior compartment of the urinary bladder and mural involving the anterior wall and extending beyond the urinary bladder. The mass measured about 7.5 × 6 × 6 cm in the mediolateral (ML) direction, anteroposterior (AP) direction, and craniocaudal (CC) direction, respectively. No perivesical lymph node enhancement was observed. Enhanced MRI of the pelvis showed a 7.5 × 6 × 6 cm right-to-left direction (RL × AP × CC) heterogeneously enhancing and slightly lobulated multifocal mass lesion, with multiple feeding vessels seen in the left anterior wall and extending into the urinary bladder and the pelvic cavity with an irregular outer margin.

**Figure 2:**
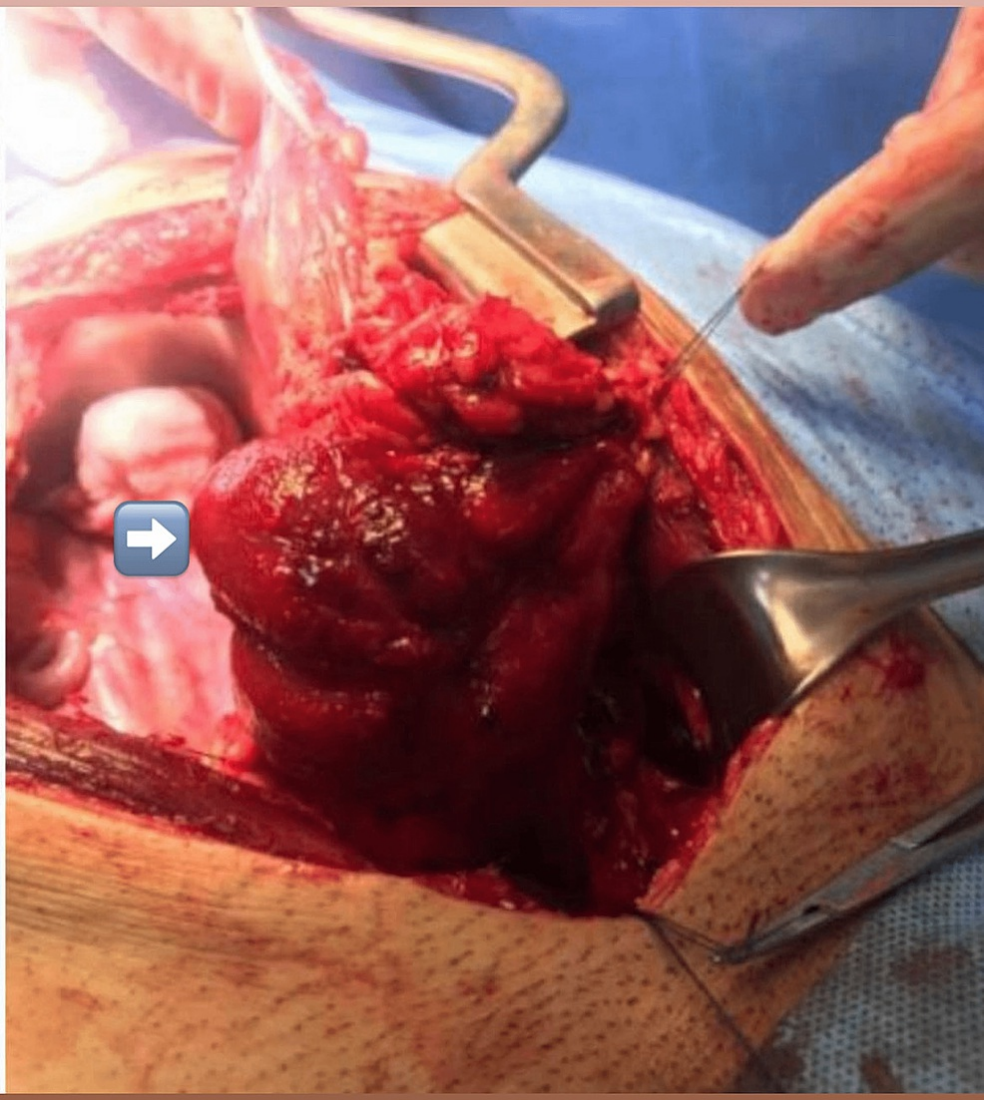
A clear view of the mass intraoperatively before resection.

**Figure 3:**
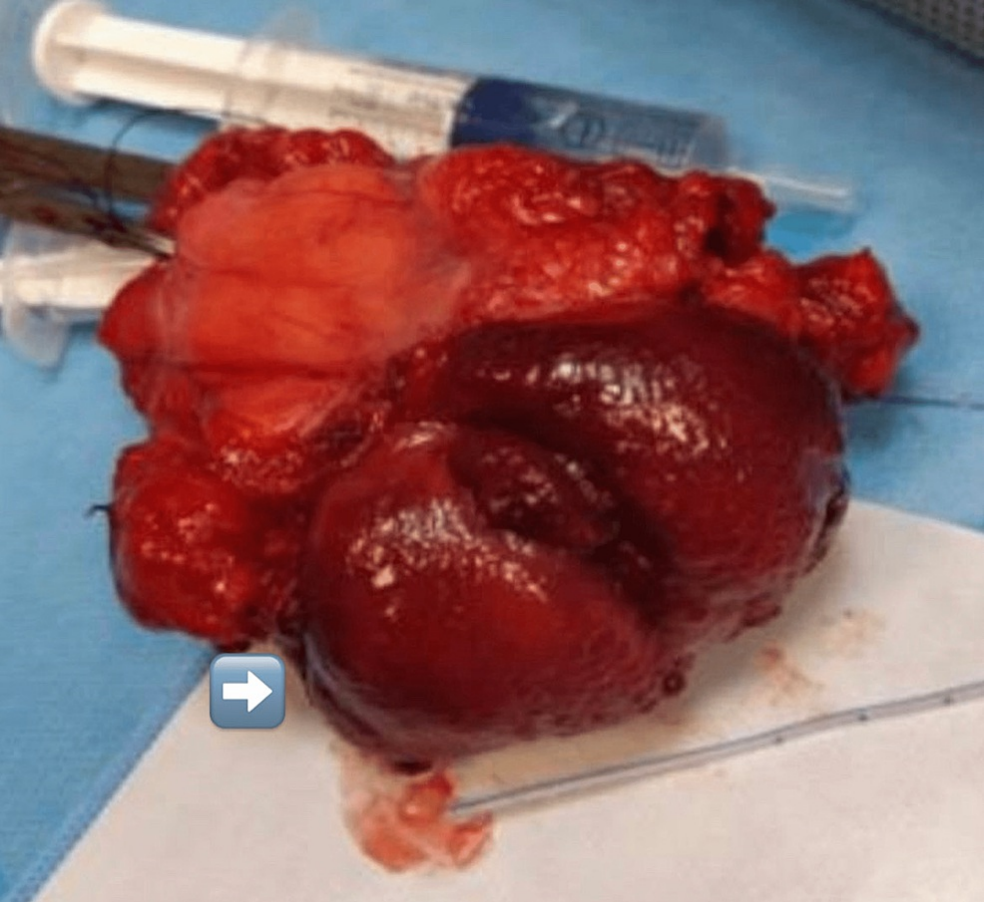
The 7 cm resected tumor with partial cystectomy.

With the assessment of the size and location of the lesion and extension, the decision was made to proceed with open partial cystectomy without augmentation. Partial cystectomy was done, and approximately one-third of the left bladder wall was removed after maintaining a safety margin. The histopathological examination of the specimen revealed cavernous hemangioma, measuring 7 cm in the MD direction, involving the lamina propria, muscularis propria, and per vesical fatty tissue (Figures 2, 3). The postoperative period was without complications. The remaining part of the bladder showed adequate urinary bladder volume, with the absence of hematuria or any mass lesions during the 20-month follow-up with cystoscopy and ultrasound. There are no standard guidelines for management and adequate follow-up period.

## Discussion

Hemangiomas are benign tumors formed by capillaries and blood vessels. The most common hemangioma in the bladder is the cavernous type, with the capillary or arteriovenous types seen less frequently [6].

Because of the lack of standard guidelines for treatment, management options are variable and unclear, including partial cystectomy for lesions measuring >3 cm or complete cystectomy with possible bladder augmentation for large masses. Several minimally invasive approaches are available for lesions measuring <3 cm. Numerous therapeutic approaches are available, including transurethral resection and electrocoagulation, partial or complete cystectomy, sclerosing agent injection, irradiation, systemic steroids, interferon-alpha-2 therapy, and, more recently, YAG-laser therapy [7]. Currently, cystoscopy is preferred to confirm the diagnosis and treat the lesion utilizing laser photocoagulation [3].

In our case, partial cystectomy was successfully done for a 7 cm mass, involving the lamina propria, muscularis propria, and perivesical fatty tissue. Management for bladder hemangioma depends on the high possibility of bleeding. When the lesion is small (measuring less than 3 cm), biopsy and fulguration are effective for hemangioma of the bladder [2].

Partial cystectomy is considered effective for masses measuring >3 cm. We aim to highlight the importance and significance of the histopathological examination of the lesion from multiple locations to define the definite subtype taken from the vascularized areas, which can guide management and prognosis. There is no evidence-based data about the recurrence and the possibility of relapse. Moreover, there are no general guidelines or protocols for an adequate follow-up period.

## Conclusions

Hemangiomas of the urinary bladder are rare and benign, and histopathological examination of tissue biopsy remains the gold standard for the diagnosis of any bladder lesion. Management options are variable and depend on the tumor size, location, and depth of invasion. Partial cystectomy is the best option for huge tumors with possible augmentation. While CHB is classified as a benign tumor, follow-up for recurrence and relapse is obligatory.
